# STIM and Orai isoform expression in pregnant human myometrium: a potential role in calcium signaling during pregnancy

**DOI:** 10.3389/fphys.2014.00169

**Published:** 2014-05-06

**Authors:** Evonne C. Chin-Smith, Donna M. Slater, Mark R. Johnson, Rachel M. Tribe

**Affiliations:** ^1^Division of Women's Health, Women's Health Academic Centre, King's College London, King's Health PartnersLondon, UK; ^2^Physiology and Pharmacology, Faculty of Medicine, University of CalgaryCalgary, AB, Canada; ^3^Academic Department of Obstetrics and Gynecology, Chelsea and Westminster Hospital, Imperial College LondonLondon, UK

**Keywords:** calcium channel, STIM, Orai, uterus, pregnant, myometrium

## Abstract

Store-operated calcium (Ca^2+^) entry (SOCE) can be mediated by two novel proteins, STIM/Orai. We have previously demonstrated that members of the TRPC family, putative basal and store operated calcium entry channels, are present in human myometrium and regulated by labor associated stimuli IL-1β and mechanical stretch. Although STIM and Orai isoforms (1-3) have been reported in other smooth muscle cell types, there is little known about the expression or gestational regulation of STIM and Orai expression in human myometrium. Total RNA was isolated from lower segment human myometrial biopsies obtained at Cesarean section from women at the time of preterm no labor (PTNL), preterm labor (PTL), term non-labor (TNL), and term with labor (TL); primary cultured human uterine smooth muscle cells, and a human myometrial cell line (hTERT-HM). STIM1-2, and Orai1-3 mRNA expression was assessed by quantitative real-time PCR. All five genes were expressed in myometrial tissue and cultured cells. STIM1-2 and Orai2-3 expression was significantly lower in cultured cells compared tissue. This has implications with regard investigation of the contribution of these proteins in cultured cells. Orai2 was the most abundant Orai isoform in human myometrium. Expression of STIM1-2/Orai1-3 did not alter with the onset of labor. Orai1 mRNA expression in cultured cells was enhanced by IL-1β treatment. This novel report of STIM1-2 and Orai1-3 mRNA expression in pregnant human myometrium and Orai1 regulation by IL-1β indicates a potential role for these proteins in calcium signaling in human myometrium during pregnancy.

## Introduction

Intracellular calcium signals regulate a variety of important physiological processes including gene transcription, cell growth, and contraction. Such signals originate either from an influx of calcium from the extracellular space [e.g., voltage-gated calcium channels, store-operated calcium entry (SOCE) and receptor-operated calcium entry (ROCE) pathways] and/or via release from intracellular calcium stores (Putney, 1986, 2013; Parekh and Penner, 1997).

In excitable cells, including myometrium, calcium entry via voltage-gated calcium channels form the predominant calcium entry pathway involved in contraction, but there is evidence for functional SOCE and ROCE in myometrial tissue and cells (Sanborn, 1995, 2007a,b; Tribe, 2001; Dalrymple et al., 2004, 2007; Shmygol and Wray, 2004; Noble et al., 2009). We hypothesized that these pathways may not only contribute to prolongation of contraction events, but also to uterine smooth muscle growth and provision of discrete calcium signals required to modulate gene expression throughout gestation and at labor onset.

It was originally proposed that transient receptor potential cation channel subfamily C (TRPC) channels contribute to SOCE and ROCE (Birnbaumer et al., 1996; Zhu et al., 1996). Indeed, we and others have identified TRPC homologues in human myometrium from non-pregnant and pregnant women (Dalrymple et al., 2002; Yang et al., 2002; Ku et al., 2006). Furthermore, we have also shown that TRPC3 expression is up-regulated by mechanical strain and the pro-inflammatory cytokine IL-1β, stimulators of myometrial growth and activation, in cultured human myometrial cells (Dalrymple et al., 2004, 2007).

However, two other gene families, STIM (1-2) and Orai (1-3) have emerged as the main candidates underlying SOCE complexes (Manji et al., 2001; Williams et al., 2001; Marchant, 2005; Roos et al., 2005; Zhang et al., 2005; Peinelt et al., 2006; Spassova et al., 2006; Soboloff et al., 2006). STIM1 senses Ca^2+^ store depletion of the endoplasmic reticulum (ER) and relocates to the plasma membrane to interact with Orai1 channels and activate Ca^2+^ entry (Zhang et al., 2005). The functional role of STIM2 is less well defined. Knockdown of STIM2 in several cell types has little or no effect on SOCE (Liou et al., 2005; Roos et al., 2005; Soboloff et al., 2006; Wu et al., 2006; Oh-Hora et al., 2008), although STIM2 has also been reported to be involved in the regulation of basal and cytosolic and ER Ca^2+^ levels (Brandman et al., 2007; Lu et al., 2009).

Orai proteins have been firmly established as the Ca^2+^-selective pores of CRAC channels. (Feske et al., 2006; Prakriya et al., 2006; Soboloff et al., 2006; Vig et al., 2006; Fahrner et al., 2013), but as SOC currents display distinctly different characteristics in excitable cells, it has yet to be fully established whether Orai channels or STIM can also mediate this more non-selective cation current alone or whether interactions between Orai1 and TRPC1/TRPC3 (Zbidi et al., 2009; Cheng et al., 2011) and/or post-translational modification of Orai may also mediate altered channel selectivity in excitable cells.

Peel et al. (2006, 2008) have demonstrated a contribution of STIM1 and Orai1 to non-selective SOC currents in cultured human airway smooth muscle cells. There is also evidence for Orai and STIM in other smooth muscle cell types (Trebak, 2012; Ruhle and Trebak, 2013). In light of this emerging role of Orai and STIM in excitable cells, the aims of the current study were to: (i) determine the mRNA expression profiles of STIM1-2 and Orai1-3 and in pregnant human myometrial tissue and primary cultured human uterine smooth muscle cells and (ii) examine the effect of the labor-associated cytokine IL-1β on these genes.

## Materials and methods

### Materials

STIM1 and β-actin antibodies were purchased from Abcam (Cambridge, UK). Orai1 antibody was purchased from Axxora (Nottingham, UK.) HRP conjugated goat anti-rabbit IgG was purchased from Pierce (Chester, UK). HRP conjugated goat anti-mouse IgG was purchased from BD Transduction Laboratories (Cowley, UK). ECL western blotting detection reagent and Hyperfilm ECL were obtained from GE Healthcare Life Sciences (Buckinghamshire, UK). SDS buffer was purchased from National Diagnostics (Hull, UK). SensiMix Plus SYBR was purchased from Bioline (London, UK). All other materials were purchased from Sigma (Poole, UK) or Invitrogen (Paisely, UK).

### Subjects

Human myometrial biopsies were obtained from women without any underlying conditions, undergoing elective Cesarean section at term (39.7 ± 0.4 weeks of gestation) with informed written consent and institutional Ethics Committee approval (St Thomas' Research Ethics Committee – EC001/137; Office of Medical Bioethics, University of Calgary). In addition, lower segment human myometrium was obtained from four groups of women at the time of Cesarean section under the conditions of preterm no labor (PTNL; 29.2 ± 1.7 weeks), preterm with labor (PTL; 30.0 ± 2.1 weeks), term no labor (TNL; 39.5 ± 0.4 weeks), and term with labor (TL; 39.1 ± 0.6 weeks). Whole myometrial tissue was either snap frozen and stored at −80°C or used immediately for cell culture. RNA was extracted from whole tissue, primary cultured myometrial cells (P0) and passaged (P2) myometrial cells and a human myometrial cell line derived from the non-pregnant uterus (hTERT-HM) (Condon et al., 2002).

### Isolation and primary culture of human uterine smooth muscle cells (hUSMCs)

hUSMCs were isolated as previously described (Tribe et al., 2000). Briefly, small segments of myometrium were dissected and chopped 1–2 mm^3^ pieces and incubated for 30–40 min in Dulbecco's modified Eagles medium (DMEM) containing 1 mg/ml collagenase 1A, 1 mg/ml collagenase XI plus 0.1% BSA, penicillin (50 units/mL), and streptomycin (50 mg/mL). Cells were dislodged using a Pasteur pipette and then filtered through a 45 μm sterile filter, and washed twice in DMEM containing 10% fetal calf serum (FCS) by centrifugation (450 × *g* 5 min). The cell pellet was suspended in DMEM supplemented with 10% FCS, penicillin (25 units/ml), and streptomycin (25 mg/ml). Primary myocytes (P0) were seeded in six-well plates and incubated at 37°C in a humidified atmosphere of 95% air/5% CO_2_. P2 myocytes were cultured in T25 flasks and then passaged in 6-well plates. After the first 2 days of culture, media was replaced with DMEM supplemented with 5% FCS, penicillin (25 units/ml), and streptomycin (25 mg/ml). The medium was changed every 2 days until cells were ~80% confluent. Cells were then passaged to P2 or serum-deprived for 24 h prior to RNA/protein extraction.

### Quantitative RT-PCR

Frozen myometrial tissue (~30 mg) was homogenized using a Qiagen Tissue Lyser. Total RNA was extracted from myometrial tissue and cultured uterine myocytes using the RNeasy mini kit (Qiagen, UK) according to the manufacturer's instructions. Complementary deoxyribonucleic acid (cDNA) was synthesized using an Omniscript RT Kit (Qiagen, UK). Real-time polymerase chain reaction (PCR) was carried out with the use of SYBR Green chemistry (Bioline) on a RotorGene 6000 (Qiagen, UK) using the primers as listed in Table 1. A pre-PCR cycle was run for 10 min at 95°C followed by 35 cycles of 95°C for 15 s, 60°C for 30 s, and 72°C for 50 s followed by a final extension at 72°C for 15 s. Melt curve analysis was performed to confirm the presence of one single product. Cycle threshold (CT) values were used for analysis, and abundance data were obtained by the use of quantified cDNA to generate a standard curve. All unknowns fell within the dynamic range of the standard curve. Standards were quantified using densitometry, and tenfold serial dilution from 10^10^ to 10^1^ copies was run in parallel with the samples. Data for the genes of interest were then expressed relative to glyceraldehyde-3-phosphate dehydrogenase (GAPDH) which was the most stable housekeeper from a panel of 3 (GAPDH, β-actin and β-2 microglobulin). All PCR products were sequenced to confirm identity.

**Table 1:** **Real-time PCR primer sequences**.

**Gene**	**Accession number**	**Primer sequence**	**Amplicon length**
STIM1	NM_003156.2	(+) 5′-GCTCCTCTGGGGACTCCT-3′	125 bp
		(−) 5′-CAATTCGGCAAAACTCTGCT-3′	
STIM2	NM_020860.1	(+) 5′-GAC GTC AGT ATG CAG AAC AGG A-3′	125 bp
		(−) 5′-TCA AAT TCT TTT TCG GCC TTT-3′	
Orai1	NM_032790.2	(+) 5′-ACC TCG GCT CTG CTC TCC-3′	147 bp
		(−) 5′-GAT CAT GAG CGC AAA CAG G-3′	
Orai2	NM_032831.1	(+) 5′-TAC CTG AGC AGG GCC AAG-3′	109 bp
		(−) 5′-GGT ACT GGT ACT GCG TCT CAA-3′	
Orai3	NM_152288.2	(+) 5′-ACG TCT GCC TTG CTC TCG-3′	141 bp
		(−) 5′-GAG TGC AAA GAG GTG CAC AG-3′	
PTGS2	NM_000963.2	(+) 5′-TCA CGC ATC AGT TTT TCA AGA-3′	94 bp
		(−) 5′-TCA CCG TTA ATA TGA TTT AAG TCC AC-3′	
GAPDH	NM_002046.3	(+) 5′-GGAAGCTTGTCATCAATGGAA-3′	102 bp
		(−) 5′-TGGACTCCACGACGTACTCA-3′	

### Identification of STIM1 and Orai1 in myometrial tissue and cultured uterine smooth muscle cells by western blotting

Human myometrial tissues were homogenized using a Tissue Lyser II (Qiagen) at 25 Hz for 3 min in 20 μl lysis buffer/mg of tissue [10 mM of HEPES-KOH (pH 7); 1 mM of dithiothreitol; 1% nonident-P40, and protease-inhibitor cocktail (COMPLETE tablets, Boehringer-Mannheim Biochemical, Lewes, Sussex, UK)]. Tissue lysates were then transferred to 1.5 ml microfuge tubes and centrifuged for 1 min at 12470 × *g* to remove any tissue debris. Uterine smooth muscle cells lysates were prepared by removing culture medium and rising twice with ice-cold PBS. The cells were then aspirated to dryness and 100 μl of lysis buffer was added to each well and incubated on ice for 5 min. Cells were then scraped and the lysate removed and placed in to a 1.5 ml microfuge tube and centrifuged for 1 min at 12,470 × *g* to remove any cell debris. Tissue and whole cell lysate protein content was quantified using a NanoDrop ND-1000 spectrophotometer (Labtech). Lysates were then diluted 1:1 with × 2 Laemmli sample buffer (Sigma) then boiled at 95°C for 5 min. Samples were then stored at −20°C until required, when they were thawed, then re-boiled for 5 min at 95°C and centrifuged at 12,470 × *g* for 1 min prior to electrophoresis.

### SDS-page and western immunoblotting

Tissue/cell lysate proteins were separated using 10% Tris-Glycine precast gels (Invitrogen) using the XCell *SureLock*™ Mini-Cell system (Invitrogen). Following electrophoresis, proteins were transferred to Immobilion™-P transfer membrane (Millipore) using the XCell *SureLock*™ Mini-Cell blotting module wet transfer blotting system. After transfer of proteins to the membrane, non-specific sites were blocked by soaking the membrane in 100% methanol for 10 s and then allowing the membrane to dry completely for ~15 min. Membranes were then incubated for at least 5 h with maximum speed of agitation at room temperature or overnight at 4°C with the appropriate primary antibody diluted in tris-buffered saline with tween [TBS-T: 50 mM Tris, 150 mM NaCl, 0.2% (v/v) Tween-20, pH 7.4] containing 10% (w/v) BSA (STIM1- 1 μg/ml; Orai1-1 μg/ml). Membranes were then washed for 3 × 20 min in TBS-T. Following the incubation of the membrane in a 1:10,000 dilution in TBS-T of horseradish peroxidase (HRP)-conjugated secondary antibody for 45 min, the membrane was washed a further 3 × 20 min in TBS-T. Immunoreactive proteins were visualized using enhanced chemiluminescence (ECL™) (Amersham) according to the manufacturer's instructions. Densitometric quantification of immunoreactive bands was carried out using BioRad Molecular Quantity One software, version 4.4.0. To verify equal protein loading, blots were also probed with β-actin antibody.

### Statistical analysis

Data were analyzed by analysis of variance (ANOVA) with repeated measures, with Bonferroni multiple comparison test or Student's *t*-test as appropriate using GraphPad Prism version 5.02 (La Jolla, CA). The effect of IL-1β on gene expression was analyzed using linear regression on the log of concentration, testing for a linear interaction between treatment and time. The standard errors were adjusted using the Huber sandwich estimator (Rogers, 1993). Data expressed as mean ± standard error of the mean (s.e.m). *P* < 0.05 was accepted as significant.

## Results

### STIM1 and STIM2 are expressed in human myometrial tissue and cultured hUSMCs

**Figure 1:**
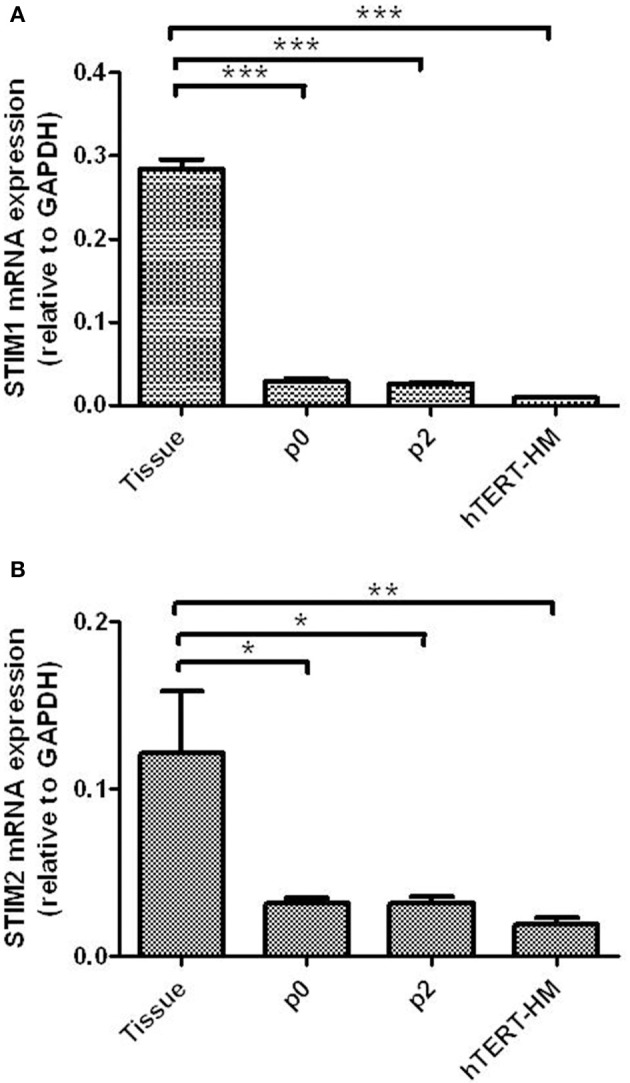
**STIM1 and STIM2 mRNA are expressed in term pregnant human myometrial tissue and cultured term pregnant (P0/P2) and non-pregnant (hTERT-HM) human uterine smooth muscle cells (hUSMCs)**. mRNA from myometrial tissue and serum-deprived hUSMCs was extracted and assessed for **(A)** STIM1 and **(B)** STIM2 expression. Data are expressed as copy number ± s.e.m., normalized to GAPDH (*n* = 6 tissues or cell cultures). ^*^P < 0.05; ^**^P < 0.01; ^***^P < 0.001 compared to tissue expression.

STIM1 mRNA was detected in myometrial tissue and cultured cells (Figure 1A). Expression was significantly higher in myometrial tissue compared to cells cultured from P0 and P2 (term-pregnant) and hTERT-HM (non-pregnant) hUSMCs. STIM1 mRNA expression in hTERT-HM uterine smooth muscle cells was significantly less than expression in P0 or P2 cells (*P* < 0.0002). STIM2 mRNA was also detected, with significantly greater expression in myometrial tissue compared to term-pregnant and non-pregnant hUSMCs (*P* < 0.05) (Figure 1B). Similarly STIM2 mRNA expression was significantly less in hTERT-HM compared to P0 and P2 uterine smooth muscle cells (*P* < 0.05).

### Orai1, Orai2, and Orai3 mRNA are expressed in human myometrial tissue and cultured hUSMCs

**Figure 2:**
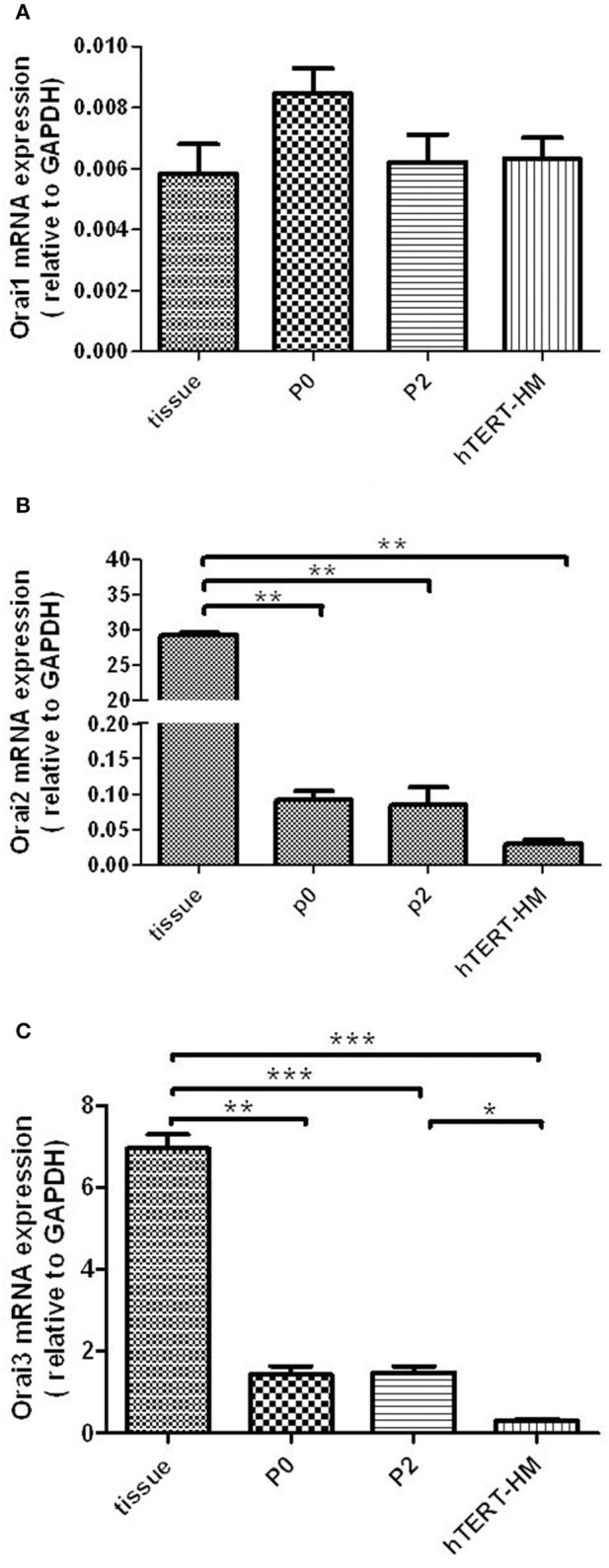
**Orai1-3 mRNA is expressed in term pregnant human myometrial tissue and cultured term pregnant (P0/P2) and non-pregnant (hTERT-HM) hUSMCs**. mRNA from myometrial tissue and serum-deprived hUSMCs was extracted and assessed for **(A)** Orai1, **(B)** Orai2, and **(C)** Orai3 expression. Data are expressed as mean ± s.e.m., normalized to GAPDH (*n* = 6 tissues or cell cultures). ^*^P < 0.05; ^**^P < 0.01; ^***^P < 0.001.

All three Orai isoforms (Orai1-3) mRNA were detected in myometrial tissue and cultured hUSMCs (Figures 2A–C). However, in contrast to STIM1 and 2 mRNA expression, Orai1 mRNA expression levels (Figure 2A), were similar in myometrial tissue and cultured cells, with no difference in expression between term-pregnant and non-pregnant hUSMCs being detected. Orai2 mRNA expression was significantly higher in tissue when compared to cultured cells (*P* < 0.01). However, expression in hTERT-HM was significantly reduced compared to P0 (*P* < 0.01) and P2 (*P* < 0.05) cultured myometrial cells. Orai3 expression was significantly higher in myometrial tissue than cultured cells with hTERT-HM exhibiting the lowest level of expression.

### Identification of STIM1 and Orai1 protein in myometrial tissue and cultured hUSMCs

**Figure 3:**
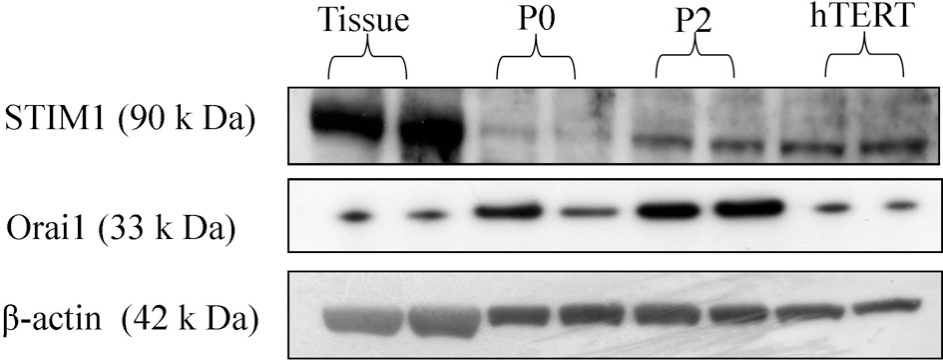
**STIM1 and Orai1 protein expression in human myometrium and cultured hUSMCs**. Human myometrial tissue and whole cell lysates proteins were separated by SDS-PAGE and probed for STIM1, Orai1, and β-actin (loading control). Representative blots are shown with each band representing tissue/cells from individual patients.

Both STIM1 and Orai1 proteins are expressed in tissue as well as cultured cells (Figure 3).

### STIM1/2 and Orai1-3 mRNA expression in pregnant human myometrial tissue

**Figure 4:**
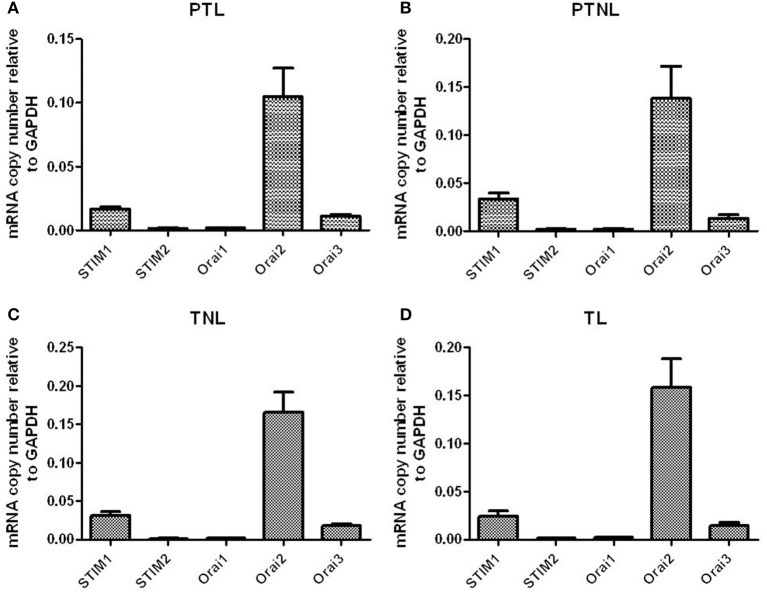
**STIM1/2 and Orai1-3 mRNA expression in pregnant human myometrial tissue taken from women at Cesarean section who were (A) preterm in labor (PTL, *n* = 4), (B) preterm but not in non labor (PTNL, *n* = 5), (C) term but not in labor (TNL, *n* = 6) and (D) term in labor (TL, *n* = 6)**.

All genes were expressed in both preterm and term myometrial tissue. There was no difference in the expression of these genes across gestation or with the onset of labor. Expression was in the order of STIM1 > STIM2 and Orai2 > Orai3 > Orai1 in all groups studied (Figures 4A–D).

### Effect of IL-1β on STIM1-2 and Orai1-3 mRNA expression in primary hUSMCs

**Figure 5:**
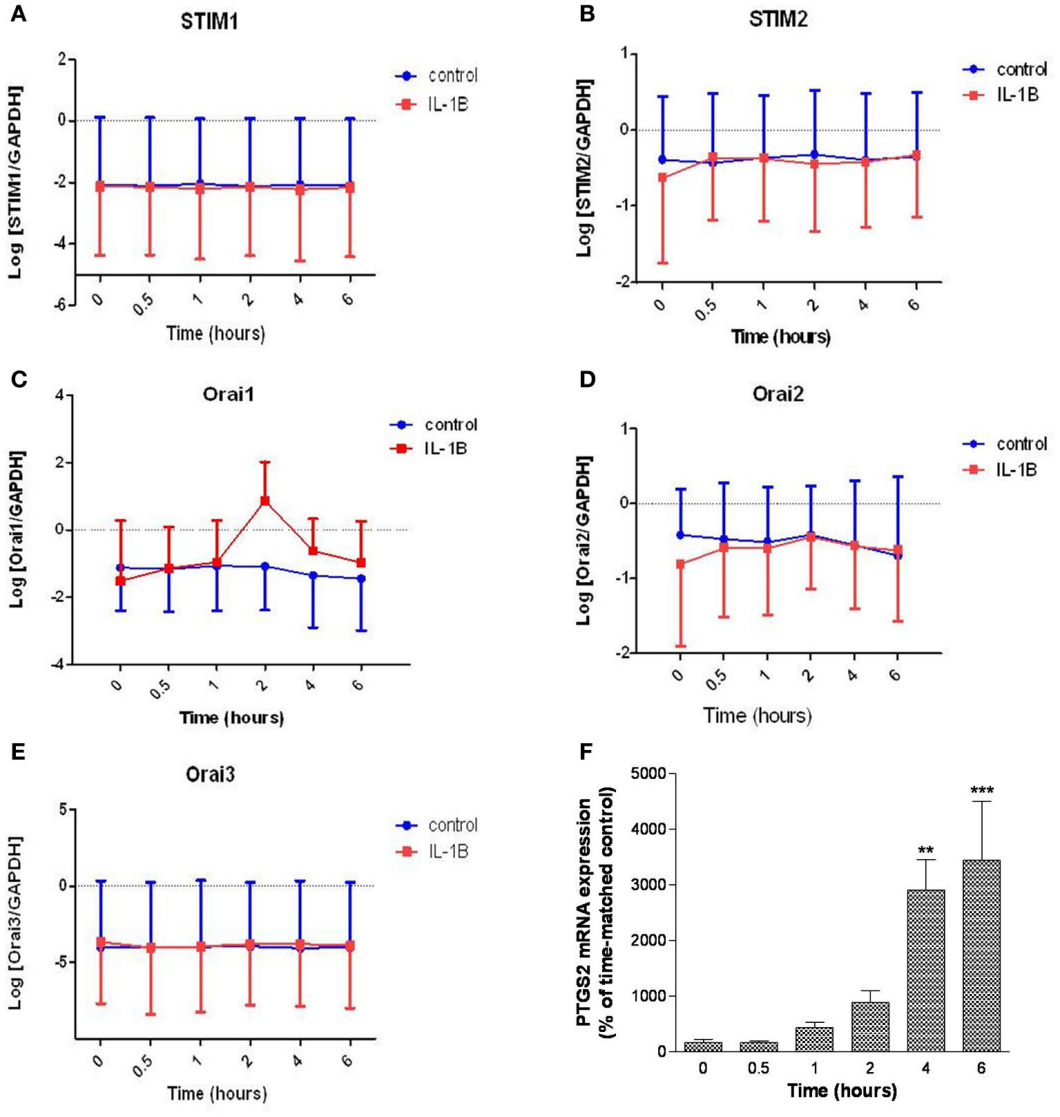
**Effect of IL-1β on STIM1-2 and Orai1-3 mRNA expression in primary hUSMCs**. Serum-deprived primary hUSMCs were incubated in the presence or absence of 10 ng/ml IL-1β for up to 6 h and mRNA extracted and assessed for **(A)** STIM1, **(B)** STIM2, **(C)** Orai1, **(D)** Orai2, **(E)** Orai3, and **(F)** PTGS2 expression. Data are expressed as log normalized copy number **(A–E)** or percentage of time-matched control **(F)**, mean ± s.e.m., (cells from *n* = 6 patients). ^**^P < 0.01; ^***^P < 0.001.

The impact of the labor-associated cytokine IL-1β treatment on STIM1-2 and Orai1-3 mRNA expression levels was also assessed (Figure 5). Six hours treatment with IL-1β (10 ng/ml) did not modulate STIM1-2 mRNA expression (Figures 5A,B). IL-1β treatment increased Orai1 mRNA expression (1.4-fold per hour up to 2 h; 95% CI: 1.1–1.7; *P* = 0.013) (Figure 5C). IL-1β had no effect on either Orai2 or Orai3 mRNA expression (Figures 5D,E). Prostaglandin-endoperoxide synthase 2 (PTGS2) mRNA expression (positive control) was significantly increased at 4 and 6 h incubation from the same experiment (Figure 5F).

### Effect of IL-1β on Orai1 protein expression in primary hUSMCs

**Figure 6:**
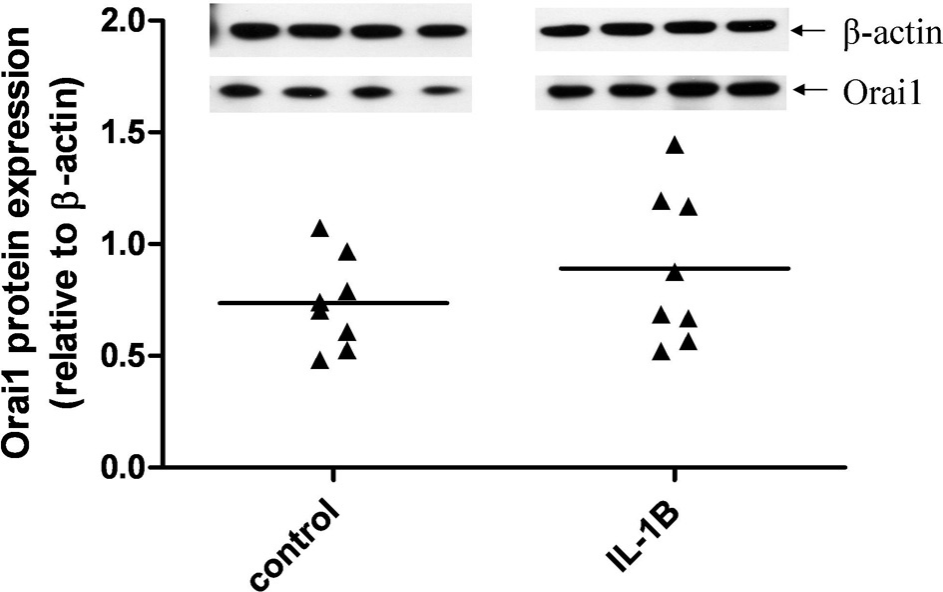
**Effect of IL-1 β on Orai1 protein expression in primary hUSMCs**. Serum-deprived primary hUSMCs were incubated in the presence or absence of 10 ng/ml IL-1β for 24 h. Whole cell lysates were assessed for Orai1 protein expression. Data are expressed relative to b-actin protein expression, (cells from *n* = 8 patients).

Given that IL-1β significantly enhanced Orai1 mRNA expression, we also investigated the effect of this cytokine on Orai1 protein expression in primary hUSMCs. Although the data did not achieve statistical significance, 7 out of 8 patients showed an increase in Orai1 expression with IL-1β (Figure 6). However, there was great inter-subject variation in the magnitude of the response to IL-1β.

## Discussion

In the present study, we have clearly demonstrated that STIM and Orai isoforms are expressed in pregnant human myometrial tissue. We show for the first time, STIM (1-2) and Orai (1-3) gene expression in pregnant preterm and term human myometrial tissue taken prior to and after the onset of labor. The identification of these genes supports previous observations of their presence in cultured human myometrial cells (Murtazina et al., 2011) and other smooth muscle cell types (Peel et al., 2006, 2008; Berra-Romani et al., 2008; Trebak, 2012; Ruhle and Trebak, 2013) and their role in SOCE (Soboloff et al., 2006; Muik et al., 2009; Yuan et al., 2009).

Assessment of mRNA abundance indicated that STIM1 expression is greater than STIM2 in term-pregnant myometrial tissue and cultured cells. These data are similar to a previous report of STIM1 and 2 mRNA expression in cultured human myometrial cells and immortalized smooth muscle cells derived from term pregnant human myometrium (Murtazina et al., 2011). The presence of STIM1 protein in tissue and cultured cells was confirmed by Western blot. These data confirm that STIM1 is the more abundant isoform in human myometrium tissue and cells.

In myometrial tissue, we identified an Orai mRNA profile of Orai2 > Orai3 > Orai1 irrespective of gestation or labor. Interestingly, this pattern of expression was slightly modified in cultured hUSMCs (Orai3 > Orai2 > Orai1) and overall there was greater expression of Orai2-3 in tissue samples compared to cells. The switch in isoform dominance between tissue and primary cultured cells could indicate that there is contribution to the Orai1-3 signal in tissue from other cell types such as mast cells (Ashmole et al., 2013) or that there has either been a phenotypic change in culture. The significant changes observed in Orai isoform expression in cultured cells compared to tissue should be taken into consideration for future investigations into the role of these proteins in cultured cells. Orai2 and 3 appear to have distinct functional properties compared to Orai1. Much less is known about the “true” functional relevance of Orai2 and 3 (Hoth and Niemeyer, 2013). However, a recent study by Bandara et al. (2013) provides evidence that Orai2 functions as a Ca^2+^ leak channel in the ER. Orai3 has been shown to compensate for the loss of functional Orai1 (DeHaven et al., 2007; Gwack et al., 2007; Lis et al., 2007) and more recently Orai3 has been associated with cell proliferation (Faouzi et al., 2011, 2013; Ay et al., 2013; Borowiec et al., 2014). Orai3 has also been have shown to contribute to store-independent arachidonic acid-regulated Ca^2+^ (ARC) channel activity (Mignen et al., 2008). More recently, Thompson et al. (2010) reported that Orai3 subunits in a heteropentamer are responsible for the switch in selectivity from a SOC channel to an ARC channel. There are also reports of a novel SOC channel encoded by Orai3 (Motiani et al., 2010). The significance of high Orai3 expression in cultured USMCs in relation to SOCE remains to be determined, but this observation fits with some of our preliminary evidence for an arachidonic acid-sensitive modulated calcium pathway in primary cultured USMCs (data not shown).

Orai1-3 mRNA expression profiles in our primary cultured cells (P0 and P2) conflicts with that reported by Murtazina et al. (2011). There data suggested that that Orai1 was the predominant transcript in cultured pregnant myometrial cells (P3-P9) and immortalized cells derived from pregnant and non-pregnant human myometrium (Orai1 > Orai2/3). It is likely that the difference in cell culture protocols account for this. For example, Murtazina et al. (2011) maintained cells in culture for a long period of time prior to experimentation (P3-9) compared to our study (P0 and P2) and it is not clear whether cells were growth arrested prior to study.

The functional impact of differences in tissue and cell STIM and Orai isoform profiles requires consideration. Scrimgeour et al. (2009) suggest that differential expression of STIM and Orai proteins can result in altered CRAC currents (I_CRAC_). The same study suggested that cells expressing a low Orai1: STIM1 ratio produced I_CRAC_ with strong fast Ca^2+^ dependent inactivation whilst cells expressing a high Orai1:STIM1 ratio produce I_CRAC_ with strong activation and negative potentials. Murtazina et al. (2011) showed that STIM1 and Orai1-3 knockdown attenuated cyclopiazonic acid (CPA) and oxytocin-induced calcium entry knockdown in immortalized and primary cultured human myometrial cells. These data supports a functional role for STIM and Orai in SOCE in human myometrium.

We also detected differences in the Orai: STIM mRNA ratio in human pregnant tissue compared to cells. Notably in our study, the endogenous Orai1:STIM1 ratio was low in primary cultured cells (P0 ~1:35; P2 ~1:43) compared to pregnant human myometrial tissue (1:10) indicating the likelihood of different SOCE currents in native tissue vs. cells maintained in culture. In non-pregnant hUSMCs the ratio was different again (~1:1.5); higher to that in cells originating from pregnant women.

Previously, we reported that IL-1β induced a significant enhancement of basal calcium entry and SOC, in tandem with TRPC3, in primary hUSMCs. However, only Orai1 was increased by IL-1β treatment. IL-1β induced a significant transient increase in Orai1 mRNA which was in most tissues associated with a rise in protein expression. A differential enhancement of Orai1 protein could in theory shift the STIM1:Orai1 ratio and potentially calcium current characteristics. However, it must be noted that a rise in Orai1 mRNA expression was not detected in tissues from women pre and post labor onset; this potentially suggests that inflammation does not influence expression of this isoform *in vivo*.

In summary, we demonstrate the presence of novel proteins (STIM/Orai) in pregnant human myometrium (preterm and term) indicating a potential involvement in SOC entry in these tissues. STIM1 and Orai2 appear to be the dominant isoforms in pregnant human myometrium. The observation that IL-1β increased Orai1 mRNA expression warrants further investigation of the functional impact of this isoform. The importance of these specific proteins to human myometrial contractility remains to be determined.

## Author contributions

Evonne C. Chin-Smith and Rachel M. Tribe designed and planned the study, analyzed and interpreted the data and drafted the manuscript. Donna M. Slater collected myometrial tissue, isolated RNA and made cDNA used in the study and assisted with data interpretation. Mark R. Johnson contributed to planning of the study, interpretation of data and drafting of the manuscript.

## Funding

This study was funded by the BBSRC [BB/E011772] and Tommy's Charity [Reg Charity No. 208701].

### Conflict of interest statement

The authors declare that the research was conducted in the absence of any commercial or financial relationships that could be construed as a potential conflict of interest.
